# Interpretable Machine Learning Model for Predicting Postpartum Depression: Retrospective Study

**DOI:** 10.2196/58649

**Published:** 2025-01-20

**Authors:** Ren Zhang, Yi Liu, Zhiwei Zhang, Rui Luo, Bin Lv

**Affiliations:** 1 Department of Gynecology and Obstetrics, West China Second University Hospital, Sichuan University, Key Laboratory of Birth Defects and Related Diseases of Women and Children (Sichuan University), Ministry of Education, Chengdu, China; 2 West China School of Medicine, Sichuan University, Chengdu, China; 3 Department of Thoracic Surgery and Institute of Thoracic Oncology, West China Hospital, Sichuan University, Chengdu, China; 4 College of Engineering, University of California, Berkeley, CA, United States

**Keywords:** postpartum depression, machine learning, predictive model, risk factors, XGBoost, extreme gradient boosting, PPD

## Abstract

**Background:**

Postpartum depression (PPD) is a prevalent mental health issue with significant impacts on mothers and families. Exploring reliable predictors is crucial for the early and accurate prediction of PPD, which remains challenging.

**Objective:**

This study aimed to comprehensively collect variables from multiple aspects, develop and validate machine learning models to achieve precise prediction of PPD, and interpret the model to reveal clinical implications.

**Methods:**

This study recruited pregnant women who delivered at the West China Second University Hospital, Sichuan University. Various variables were collected from electronic medical record data and screened using least absolute shrinkage and selection operator penalty regression. Participants were divided into training (1358/2055, 66.1%) and validation (697/2055, 33.9%) sets by random sampling. Machine learning–based predictive models were developed in the training cohort. Models were validated in the validation cohort with receiver operating curve and decision curve analysis. Multiple model interpretation methods were implemented to explain the optimal model.

**Results:**

We recruited 2055 participants in this study. The extreme gradient boosting model was the optimal predictive model with the area under the receiver operating curve of 0.849. Shapley Additive Explanation indicated that the most influential predictors of PPD were antepartum depression, lower fetal weight, elevated thyroid-stimulating hormone, declined thyroid peroxidase antibodies, elevated serum ferritin, and older age.

**Conclusions:**

This study developed and validated a machine learning–based predictive model for PPD. Several significant risk factors and how they impact the prediction of PPD were revealed. These findings provide new insights into the early screening of individuals with high risk for PPD, emphasizing the need for comprehensive screening approaches that include both physiological and psychological factors.

## Introduction

Postpartum depression (PPD) is a common mental disorder characterized by low mood, loss of pleasure, and sleep disturbance during the postpartum period [1]. The prevalence of PPD ranges from 3% to 38% in different nations and is higher in limited-income countries [2,3]. PPD leads to adverse consequences for the mother and family members, such as emotional strain and increased caregiving burden. Women with PPD may experience prolonged periods of distress and are more vulnerable to recurrent depressive episodes [4]. Previous studies revealed that PPD can impair a mother’s parenting ability, such as breastfeeding, potentially resulting in enduring adverse effects on the child’s development across emotional, cognitive, and physical domains [5,6]. Moreover, PPD can strain family relationships and impose economic burdens due to increased health care needs and reduced productivity [6].

With such a profound impact, mothers should be routinely screened for PPD, and early interventions should be implemented. However, current screening for PPD is mainly based on existing depressive symptoms such as fatigue and sleep disturbance, which are believed to be overlooked due to overlap with normal physiological manifestations after delivery [7,8]. In addition, the diagnosis of PPD depends on patients’ subjective reporting of personal health conditions [9]. It is urgent to identify individuals with high risk for PPD before clinical symptoms appear, while no effective and validated screening tools are currently available [7,10].

Previous studies have identified several risk factors of PPD such as unplanned pregnancy, lack of social support, and family history of mental disorders [11,12]. However, limited variables in such studies led to a lack of integrity. Machine learning algorithms provide support for the development of predictive models to prevent and intervene adverse health outcomes, offering avenues for personalized prediction and intervention strategies [13,14]. Several studies have adapted machine learning into the prediction of PPD risk in the last few years and achieved impressive performance [15-17]. However, insufficient model explanations leave obstacles for actual implementation. Besides, mental disorders are strongly associated with cultural backgrounds and study populations. Thus, the challenge remains to develop more nuanced and culturally adaptable machine learning models for the early detection and effective management of PPD, bridging the gap in current research and practice.

Given the importance of early screening for PPD and the limitations mentioned earlier, we conducted a retrospective study at our institution. This study comprehensively collected variables from multiple aspects, adopted machine learning algorithms to identify risk factors, and aimed to achieve precise prediction of PPD.

## Methods

### Participants

Pregnant women who underwent perinatal examinations and delivered at West China Second University Hospital, Sichuan University, from January 2017 to December 2020 were invited to participate in this study. The study cohort was divided into training and validation sets by random sampling. Participants were screened for eligibility. The inclusion criteria were as follows: (1) participants who completed regular examinations and delivered at our institution, (2) participants with a gestational age of ≥28 weeks, and (3) participants who gave consent to participation and be followed up. The exclusion criteria were (1) participants with a psychiatric history in the 6 months before conception and (2) participants with missing data.

### Outcome

Participants were assessed for PPD 3 months post partum with the Edinburgh Postnatal Depression Scale [18]. The Edinburgh Postnatal Depression Scale has 10 items concerning depressive symptoms, and each item is evaluated using scores ranging from 0 to 3, constituting a total score of 30. Participants who scored 13 or more were regarded as having PPD [18]. The diagnosis of PPD was confirmed by 2 experienced senior psychiatrists using the Structured Clinical Interview for *Diagnostic and Statistical Manual of Mental Disorders, Fifth Edition* (*DSM-5*) [19] and the *Chinese Classification and Diagnostic Criteria of Mental Disorders, Third Edition* (*CCMD-3*) [20].

### Variable Screening

Demographic variables were collected from the electronic medical record system of our institution. Clinical variables were assessed and documented by qualified clinicians. Relevant laboratory indicators were collected at 28 weeks of gestation from the medical laboratory system of the institution.

Participants were assessed for antepartum depression before delivery with the Zung Self-Rating Depression Scale [21]. The Self-Rating Depression Scale is a self-reported scale with 20 items concerning depressive symptoms, and each item is evaluated with scores ranging from 0 to 4, according to the severity of symptoms. All participants with more than 53 points were regarded as having antepartum depression [22].

Social variables, including education, income, exposure to suspected adverse factors, and family and social relations, were collected using scales and self-administered questionnaires. Income level was assessed using the local minimum income standard. Suspected adverse factors included alcohol consumption and smoking. Family and social relations comprised spouses in good health, only child, planned pregnancy, social support, family satisfaction, adverse marital status, and family history of mental illness. The level of social support was measured using the Social Support Rating Scale, which is widely used to assess social support with great reliability [23]. Scores higher than 35 are considered normal; scores of 35 or lower indicate low levels of social support [24]. Family satisfaction was assessed using the Family Adaptation, Partnership, Growth, Affection, Resolve index [25]. The Family Adaptation, Partnership, Growth, Affection, Resolve index consists of 5 items, each with a score ranging from 0 to 2. It systematically evaluates the level of family care a pregnant woman receives. A total score of 0‐3 represents a low level of family satisfaction, and a score higher than 4 represents a normal level.

To avoid the potential bias of multicollinearity and overfitting, least absolute shrinkage and selection operator (LASSO) regression was performed to select and filter the variables in the training set. LASSO is a regression-based methodology that can reduce model complexity; multicollinearity and overfitting are avoided by constructing a penalty function [26]. LASSO regression is applied to filter a large number of variables and remove those that are insignificant [27,28]. The 5-fold cross-validation method was used to calculate the optimal λ values, and variables with nonzero coefficients were selected as the final predictive factors. After LASSO regression, the variance inflation factor (VIF) was calculated among the included variables to assess multicollinearity. The VIF was introduced to understand the impact of collinearity in regression models and has since been widely applied in various fields, including medical research [29-31]. VIF helps ensure that machine learning models or statistical models are not adversely affected by collinear predictors [32]. Typically, a VIF value greater than 10 is considered indicative of high multicollinearity, which may necessitate removing or adjusting variables to improve model stability [29,33].

### Model Development

We used the following 3 machine learning algorithms to develop the PPD prediction model: extreme gradient boosting (XGBoost), random forest (RF), and gradient boosting machine (GBM). XGBoost is a powerful and efficient machine learning algorithm known for its exceptional performance in regression, classification, and ranking problems. It is an extension of the traditional gradient boosting method that combines multiple weak classifiers to create a strong classifier that minimizes the loss function [34]. RF is an ensemble machine learning algorithm based on decision trees. It creates multiple decision trees, each based on a randomly sampled subset of the training data to create a more accurate and robust output [35]. GBM is a popular machine learning algorithm that combines the principles of boosting and gradient descent to create a powerful predictive model [36]. Additionally, logistic regression, a traditional method, was implemented to predict PPD as a control.

Machine learning models were developed in the training set. To mitigate overfitting and achieve ideal model performance, hyperparameters for each machine learning model were tuned by grid search. In each session of hyperparameter tuning, 3-fold cross-validation was implemented, and the area under the receiver operating characteristic curve (AUC) was the criterion to assess model performance [37]. The combination of hyperparameters with the largest AUC value was further evaluated in the validation set.

### Model Evaluation

Predictive models were evaluated with the receiver operating characteristic curve (ROC) and decision curve analysis (DCA) in the validation set. ROC reflects the ability of a model to discriminate PPD [38]. DCA is used to evaluate and compare the clinical utility of different diagnostic or predictive models. It provides a framework for assessing the net benefit of a model by taking into account the potential harms and benefits associated with different decision thresholds [39]. Additionally, accuracy, sensitivity, specificity, positive predictive value (PPV), and negative predictive value (NPV) of each model were calculated for comprehensive evaluation. Based on ROC, the predictive model with the greatest AUC value was considered as the optimal model, which would be further explored for interpretation.

### Model Interpretation

We performed variable importance, partial dependence plot (PDP), and Shapley Additive Explanation (SHAP) to interpret the optimal predictive model. Variable importance assesses the contribution of each input variable by calculating the decrease in error when split by a variable [40]. PDPs calculate the partial dependence of a variable by fixing the values of other variables and observing the variation in the outcome [41]. It helps to explain how the outcome changes with changes in input variables. SHAP measures the contribution of variables in each individual sample [42]. The SHAP values show how much each variable contributes, either positively or negatively, to the outcome.

### Statistical Software

All statistical analyses were performed with R software (version 4.3.1; R Foundation for Statistical Computing). LASSO regression was performed using the R package *glmnet*. Logistic regression model was implemented using the R package *glm*. XGBoost, RF, and GBM models were developed and assessed with R package *mlr3*. Model interpretation was performed with R packages *fastshap* and *pdp*. Other involved packages include *xgboost, randomForest, gbm, pROC, ggplot2*, and their various dependencies.

### Ethical Considerations

This study was approved by the ethics committee of West China Second University Hospital, Sichuan University (approval 2021-186). Informed consent was obtained from all individual participants involved in the study. The original informed consent covered the secondary use of the data without the need for additional consent. All participant data were anonymized to protect privacy and confidentiality. No compensation was provided for participation in this study.

## Results

### Participant Characteristics

**Figure 1.**
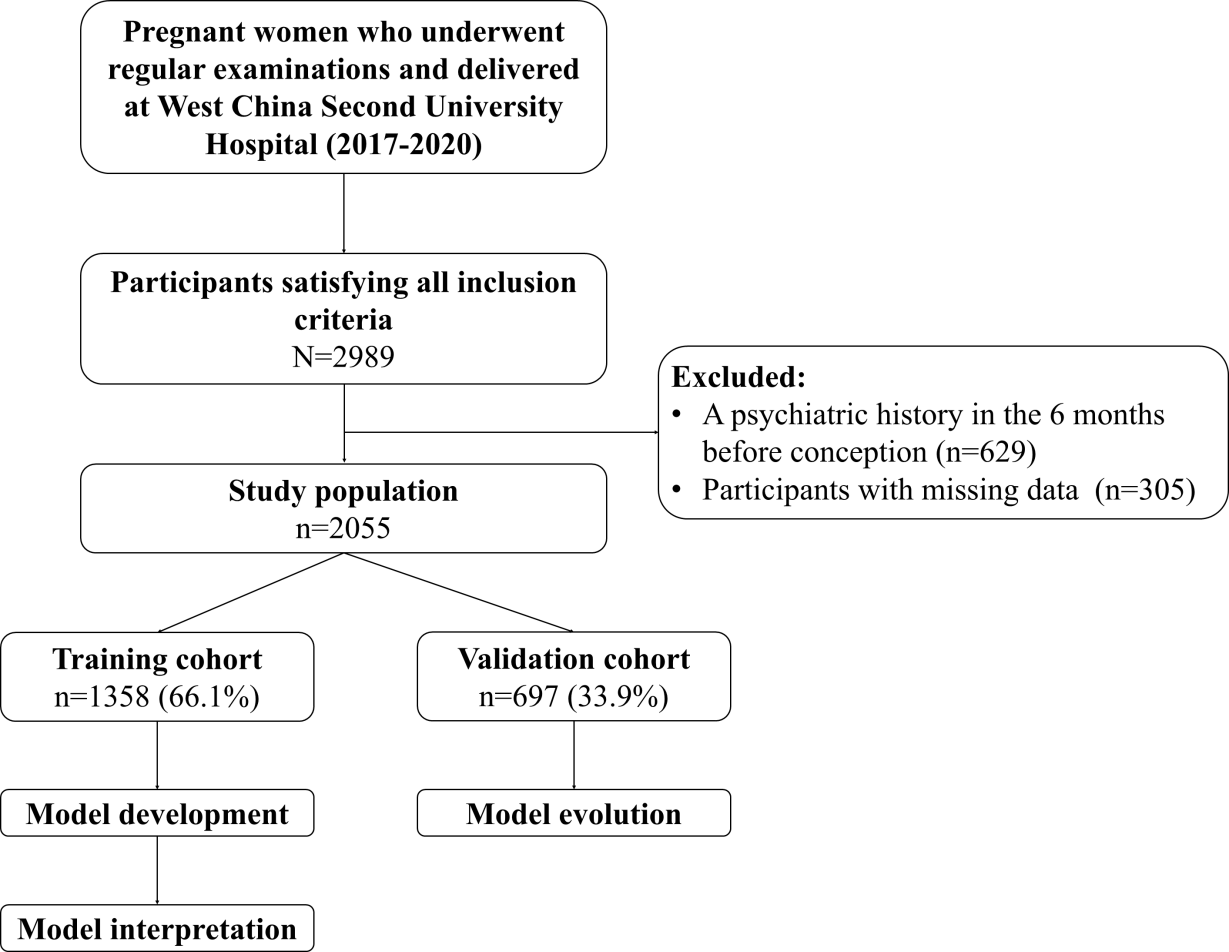
The flow chart of the study design.

The overall procedure of this study is shown in Figure 1. After eligibility screening, 2055 participants were included in the study cohort. A total of 78 variables were incorporated in our study including 16 psychosocial characteristics, 43 obstetric characteristics, and 19 laboratory indicators. The baseline characteristics were analyzed by *χ*² test and Wilcoxon test for category variables and continuous variables, respectively. The detailed characteristics are shown in Tables 1-3. Of these participants, 697 (33.9%) participants were diagnosed with PPD, 621 (30.2%) participants had antepartum depression, 101 (4.9%) were unemployed, 187 (9.1%) had an income below the local minimum income standard, 1947 (94.7%) pregnant women were the only child of the family, and 45 (2.2%) reported low family satisfaction; the median age of participants was 32 (IQR 29-35) years.

**Table 1.** Psychosocial characteristics of participants.

Psychosocial characteristics		Non-PPD^a^ (n=1358)	PPD (n=697)	*P* value	Method
Age (years), median (IQR)		31 (29-35)	32 (29-35)	.03	Wilcoxon
Antepartum depression, n (%)				<.001	*χ*² test
	No	1170 (86.2)	264 (37.9)		
	Yes	188 (13.8)	433 (62.1)		
Ethnics, n (%)				.15	*χ*² test
	Han	1326 (97.6)	673 (96.6)		
	Others	32 (2.4)	24 (3.4)		
Work status, n (%)				.002	*χ*² test
	Employed	1277 (94)	677 (97.1)		
	Unemployed	81 (6)	20 (2.9)		
Season of delivery, n (%)				.51	*χ*² test
	Spring	343 (25.3)	193 (27.7)		
	Summer	350 (25.8)	165 (23.7)		
	Autumn	411 (30.3)	217 (31.1)		
	Winter	254 (18.7)	122 (17.5)		
Education, n (%)				.63	*χ*² test
	Below higher education	459 (33.8)	243 (34.9)		
	Higher education	899 (66.2)	454 (65.1)		
Income, n (%)				.46	*χ*² test
	Below normal level	119 (8.8)	68 (9.8)		
	At or above normal level	1239 (91.2)	629 (90.2)		
Smoking, n (%)				.69	Yates’ correction
	No	1355 (99.8)	694 (99.6)		
	Yes	3 (0.2)	3 (0.4)		
Drinking, n (%)				>.99	Yates’ correction
	No	1355 (99.8)	695 (99.7)		
	Yes	3 (0.2)	2 (0.3)		
Spouse in good health, n (%)				.95	*χ*² test
	Yes	1339 (98.6)	687 (98.6)		
	No	19 (1.4)	10 (1.4)		
Only child, n (%)				.17	*χ*² test
	Yes	1280 (94.3)	667 (95.7)		
	No	78 (5.7)	30 (4.3)		
Planned pregnancy, n (%)				.31	*χ*² test
	Yes	1310 (96.5)	666 (95.6)		
	No	48 (3.5)	31 (4.4)		
Social support, n (%)				.52	*χ*² test
	Normal	1329 (97.9)	679 (97.4)		
	Low	29 (2.1)	18 (2.6)		
Family satisfaction, n (%)				.13	*χ*² test
	Normal	1333 (98.2)	677 (97.1)		
	Low	25 (1.8)	20 (2.9)		
Adverse marital status, n (%)				.006	*χ*² test
	No	1349 (99.3)	683 (98)		
	Yes	9 (0.7)	14 (2)		
Family history of mental illness, n (%)				.04	Yates’ correction
	No	1356 (99.9)	691 (99.1)		
	Yes	2 (0.1)	6 (0.9)		

a PPD: postpartum depression.

**Table 2.** Obstetric characteristics of participants.

Obstetric characteristics		Non-PPD^a^ (n=1358)	PPD (n=697)	*P* value	Method
Weight gain during pregnancy, median (IQR)		12.5 (9.425‐15)	12.5 (9.7‐16)	.39	Wilcoxon
BMI, median (IQR)		20.83 (19.43‐22.68)	20.83 (19.36‐23.01)	.63	Wilcoxon
Age of menarche (years), median (IQR)		13 (12-13)	13 (12-14)	.30	Wilcoxon
Gestational days, median (IQR)		274 (268-280)	274 (267-278)	.05	Wilcoxon
Bleeding volume, median (IQR)		400 (300-400)	400 (300-400)	.07	Wilcoxon
Fetal weight, median (IQR)		3.28 (2.94‐3.57)	3.23 (2.8‐3.53)	.001	Wilcoxon
Fetal height, median (IQR)		50 (48-51)	49 (48-51)	.03	Wilcoxon
Apgar^b^ 1 minute, median (IQR)		10 (10-10)	10 (10-10)	<.001	Wilcoxon
Apgar 5 minutes, median (IQR)		10 (10-10)	10 (10-10)	<.001	Wilcoxon
Apgar 10 minutes, median (IQR)		10 (10-10)	10 (10-10)	<.001	Wilcoxon
Length of stay, median (IQR)		4 (4-6)	4 (4-6)	.79	Wilcoxon
Gravidity				.19	*χ*² test
	1, n (%)	471 (22.9)	217 (10.6)		
	2, n (%)	370 (18)	207 (10.1)		
	3, n (%)	265 (12.9)	136 (6.6)		
	4, n (%)	160 (7.8)	79 (3.8)		
	≥5, n (%)	92 (4.5)	58 (2.8)		
	Median (IQR)	2 (1-3)	2 (1-3)		
Abortions, n (%)				.38	*χ*² test
	0	624 (45.9)	301 (43.2)		
	1	409 (30.1)	207 (29.7)		
	2	207 (15.2)	115 (16.5)		
	≥3	118 (8.7)	74 (10.6)		
Parity, n (%)				.89	Yates’ correction
	0	831 (40.4)	434 (21.1)		
	1	497 (24.2)	246 (12)		
	2	29 (1.4)	16 (0.8)		
	≥3	1 (0)	1 (0)		
Conception method, n (%)				.93	*χ*² test
	Normal	1169 (86.1)	599 (85.9)		
	Assisted reproduction	189 (13.9)	98 (14.1)		
Fetal malformation, n (%)				.02	*χ*² test
	No	1308 (96.3)	656 (94.1)		
	Yes	50 (3.7)	41 (5.9)		
Amniotic fluid volume disorder, n (%)				.35	*χ*² test
	No	1284 (94.6)	652 (93.5)		
	Yes	74 (5.4)	45 (6.5)		
Renal disease, n (%)				.58	*χ*² test
	No	1334 (98.2)	687 (98.6)		
	Yes	24 (1.8)	10 (1.4)		
Systemic lupus erythematosus, n (%)				.69	Yates’ correction
	No	1351 (99.5)	695 (99.7)		
	Yes	7 (0.5)	2 (0.3)		
Gestational diabetes mellitus, n (%)				.92	*χ*² test
	No	1030 (75.8)	530 (76)		
	Yes	328 (24.2)	167 (24)		
Gestational hypertension, n (%)				.25	*χ*² test
	No	1290 (95)	670 (96.1)		
	Yes	68 (5)	27 (3.9)		
Threatened premature labor, n (%)				.002	*χ*² test
	No	1183 (87.1)	571 (81.9)		
	Yes	175 (12.9)	126 (18.1)		
Hepatitis B, n (%)				.49	*χ*² test
	No	72 (5.3)	42 (6)		
	Yes	1286 (94.7)	655 (94)		
Twin pregnancy, n (%)				.07	*χ*² test
	No	1247 (91.8)	623 (89.4)		
	Yes	111 (8.2)	74 (10.6)		
Placenta previa, n (%)				.45	*χ*² test
	No	1281 (94.3)	663 (95.1)		
	Yes	77 (5.7)	34 (4.9)		
Heart disease, n (%)				.29	*χ*² test
	No	1344 (99)	693 (99.4)		
	Yes	14 (1)	4 (0.6)		
Scarred uterus, n (%)				.81	*χ*² test
	No	342 (25.2)	179 (25.7)		
	Yes	1016 (74.8)	518 (74.3)		
Rh blood type, n (%)				.01	*χ*² test
	Positive	1350 (99.4)	685 (98.3)		
	Negative	8 (0.6)	12 (1.7)		
ABO blood type, n (%)				.63	*χ*² test
	O	491 (36.2)	241 (34.6)		
	B	335 (24.7)	179 (25.7)		
	A	423 (31.1)	211 (30.3)		
	AB	109 (8)	66 (9.5)		
Abnormal fetal position, n (%)				.92	*χ*² test
	No	1206 (88.8)	618 (88.7)		
	Yes	152 (11.2)	79 (11.3)		
Uterine myoma, n (%)				.94	*χ*² test
	No	1227 (90.4)	629 (90.2)		
	Yes	131 (9.6)	68 (9.8)		
Ovarian cyst, n (%)				>.99	Yates’ correction
	No	1349 (99.3)	693 (99.4)		
	Yes	9 (0.7)	4 (0.6)		
Umbilical cord encirclements, n (%)				.65	*χ*² test
	No	869 (64)	453 (65)		
	Yes	489 (36)	244 (35)		
Hypothyroidism, n (%)				.40	*χ*² test
	No	1130 (83.2)	590 (84.6)		
	Yes	228 (16.8)	107 (15.4)		
Pelvic anomaly, n (%)				.56	*χ*² test
	No	1346 (99.1)	689 (98.9)		
	Yes	12 (0.9)	8 (1.1)		
Intrauterine death, n (%)				<.001	Yates’ correction
	No	1358 (100)	684 (98.1)		
	Yes	0 (0)	13 (1.9)		
Macrosomia, n (%)				.84	*χ*² test
	No	1295 (95.4)	666 (95.6)		
	Yes	63 (4.6)	31 (4.4)		
Fetal growth restriction, n (%)				.34	*χ*² test
	No	1335 (98.3)	681 (97.7)		
	Yes	23 (1.7)	16 (2.3)		
Premature labor, n (%)				<.001	*χ*² test
	No	1207 (88.9)	579 (83.1)		
	Yes	151 (11.1)	118 (16.9)		
Mode of delivery, n (%)				.45	Yates’ correction
	Vaginal delivery	842 (62)	433 (62.1)		
	Cesarean section	506 (37.3)	262 (37.6)		
	Assisted delivery	10 (0.7)	2 (0.3)		
Fetal sex, n (%)				.96	*χ*² test
	Male	668 (49.2)	342 (49.1)		
	Female	690 (50.8)	355 (50.9)		
Fetal distress, n (%)				.001	*χ*² test
	No	1333 (98.2)	667 (95.7)		
	Yes	25 (1.8)	30 (4.3)		
Breastfeeding, n (%)				.61	*χ*² test
	No	32 (2.4)	19 (2.7)		
	Yes	1326 (97.6)	678 (97.3)		

a PPD: postpartum depression.

b Apgar: appearance, pulse, grimace, activity, and respiration.

**Table 3.** Laboratory indicators.

Laboratory indicators	Non-PPD^a^ (n=1358), median (IQR)	PPD (n=697), median (IQR)	*P* value	Method
Hemoglobin (g/L)	111 (104‐117.75)	111 (104-118)	.893	Wilcoxon
Serum ferroprotein (ng/nL)	18.15 (12.4‐25.9)	18.9 (11.9‐27.2)	.400	Wilcoxon
International normalized ratio	0.97 (0.92‐1.01)	0.96 (0.91‐1.01)	.325	Wilcoxon
Alanine aminotransferase (U/L)	17 (12.25‐28)	18 (12-31)	.170	Wilcoxon
Aspartate aminotransferase (U/L)	21 (18-27)	21 (17-28)	.263	Wilcoxon
Total bile acid (µmol/L)	2.3 (1.6‐3.5)	2.5 (1.6‐3.7)	.091	Wilcoxon
Direct bilirubin (µmol/L)	2.1 (1.6‐2.8)	2.1 (1.7‐2.7)	.771	Wilcoxon
Albumin (g/L)	38.7 (36.3‐41.2)	38.7 (36.3‐41.4)	.627	Wilcoxon
Globulin (g/L)	27.6 (25.4‐30.1)	27.3 (25.2‐29.9)	.207	Wilcoxon
Lactate dehydrogenase (U/L)	179 (163-201)	181 (164-204)	.161	Wilcoxon
Alkaline phosphatase (U/L)	84 (55-121)	87 (55-128)	.423	Wilcoxon
Urea nitrogen (µmol/L)	3.5 (3.07‐4.3475)	3.48 (3.08‐4.35)	.787	Wilcoxon
Creatinine (µmol/L)	44 (40-48)	44 (40-48)	.561	Wilcoxon
Cystatin C (µmol/L)	0.77 (0.64‐0.97)	0.77 (0.64‐0.99)	.725	Wilcoxon
Uric acid (µmol/L)	259 (217-309)	254 (218-305)	.250	Wilcoxon
Thyroid-stimulating hormone (mIU/L)	1.9695 (1.277‐2.8878)	1.847 (1.176‐2.776)	.183	Wilcoxon
Free thyroxine (pmol/L)	14.56 (13.29‐16.22)	14.55 (13.21‐16.02)	.522	Wilcoxon
Thyroid peroxidase antibody (U/mL)	40.65 (30.4‐56.1)	40.1 (30.2‐55.3)	.425	Wilcoxon
Vitamin D (nmol/L)	23.9 (17.3‐31.3)	22.9 (16.1‐29.8)	.011	Wilcoxon

a PPD: postpartum depression.

### Variable Screening

After LASSO regression, 18 variables with nonzero coefficients were identified as potential predictors of PPD. Among these variables, the 5-minute Apgar (appearance, pulse, grimace, activity, and respiration) score and the 10-minute Apgar score had VIF values over 10, indicating multicollinearity between them. Of these 2 variables, the 5-minute Apgar score had a lower coefficient in absolute value in the LASSO regression, suggesting its lower contribution to the outcome; therefore, it was excluded. Another round of VIF analysis was performed after excluding the 5-minute Apgar score, and the results showed that all remaining variables had a VIF below 10, indicating low multicollinearity. Finally, 17 variables including prenatal depression, ethnics, occupation, income, only child, family satisfaction, adverse marital status, amniotic fluid volume disorder, Rh negative, intrauterine death, fetal distress, age, fetal weight, 10-minute Apgar score, serum ferroprotein, thyroid-stimulating hormone (TSH), and thyroid peroxidase antibody (TPOAb) were identified as features to develop predictive models. The detailed results of the LASSO regression and VIF analyses are presented in Table 4.

**Table 4.** LASSO^a^ coefficients and VIF^b^ of screened variables after LASSO regression.

Variable	Coefficient	VIF	VIF (second round)
Prenatal depression	2.245	1.014	1.013
Ethnics	0.055	1.008	1.008
Occupation	−0.215	1.047	1.045
Income	0.015	1.043	1.043
Only child	0.081	1.013	1.012
Family satisfaction	0.134	1.287	1.286
Adverse marital status	0.422	1.296	1.293
Amniotic fluid volume disorder	0.416	1.011	1.011
Rh negative	−0.163	1.012	1.011
Intrauterine death	1.042	1.487	1.486
Fetal distress	0.339	1.034	1.034
Age	0.002	1.035	1.034
Fetal weight	−0.17	1.472	1.347
Apgar^c^ 5 minutes	−0.002	44.005	N/A^d^
Apgar 10 minutes	−0.155	42.777	1.860
Serum ferroprotein	0.001	1.043	1.043
TSH^e^	−0.001	1.003	1.003
TPOAb^f^	0.001	1.008	1.008

a LASSO: least absolute shrinkage and selection operator.

b VIF: variance inflation factor.

c Apgar: appearance, pulse, grimace, activity, and respiration.

d N/A: not applicable.

e TSH: thyroid-stimulating hormone.

f TPOAb: thyroid peroxidase antibody.

### Model Development and Evaluation

**Figure 2.**
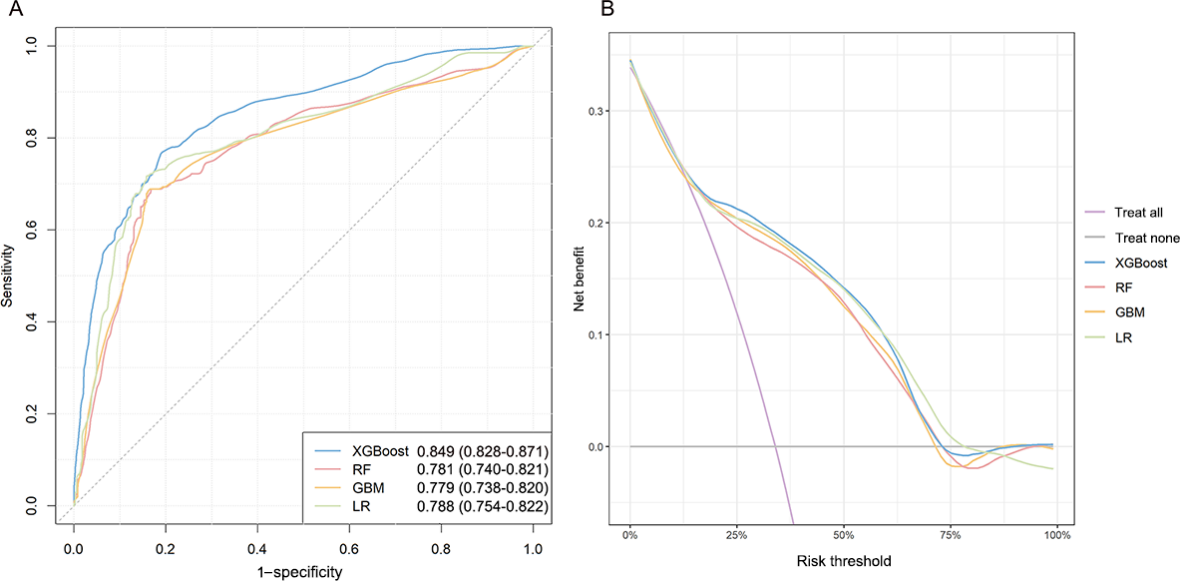
(**A**) The ROC and (**B**) the DCA of predictive models. The area under the curve and the corresponding 95% CI for each model are shown in the legend of Figure 2A. DCA: decision curve analysis; GBM: gradient boosting machine; LR: logistic regression; RF: random forest; ROC: receiver operating characteristic curve; XGBoost: extreme gradient boosting.

Predictive models were established in the training set. With optimal hyperparameters, models were evaluated in the validation set. The AUC values obtained in the validation set of all 4 models were above 0.75 (Table 5 and Figure 2A). The XGBoost model outperformed other models with the highest AUC of 0.849 (95% CI 0.828‐0.871). GBM had the poorest performance with an AUC value of 0.779 (95% CI 0.738‐0.820). Detailed results of model evaluation were shown in Table 5.

**Table 5.** Model evaluation metrics.

	AUC^a^ (95% CI)	Accuracy (95% CI)	Sensitivity (95% CI)	Specificity (95% CI)	PPV^b^ (95% CI)	NPV^c^ (95% CI)
XGBoost^d^	0.849 (0.828‐0.871)	0.813 (0.772‐0.854)	0.718 (0.646‐0.790)	0.862 (0.807‐0.917)	0.718 (0.646‐0.790)	0.855 (0.805‐0.905)
RF^e^	0.781 (0.740‐0.821)	0.782 (0.750‐0.815)	0.656 (0.591‐0.720)	0.848 (0.813‐0.883)	0.688 (0.624‐0.753)	0.827 (0.791‐0.864)
GBM^f^	0.779 (0.738‐0.820)	0.786 (0.753‐0.818)	0.675 (0.611‐0.738)	0.843 (0.807‐0.878)	0.688 (0.624‐0.751)	0.835 (0.799‐0.870)
LR^g^	0.788 (0.754‐0.822)	0.779 (0.739‐0.816)	0.685 (0.615‐0.748)	0.823 (0.782‐0.858)	0.646 (0.577‐0.710)	0.848 (0.808‐0.881)

a AUC: area under the receiver operating characteristic curve.

b PPV: positive predictive value.

c NPV: negative predictive value.

d XGBoost: extreme gradient boosting.

e RF: random forest.

f GBM: gradient boosting machine.

g LR: logistic regression.

DCA (Figure 2B) was performed for 4 models in the validation set to compare the net benefit of the best model and alternative approaches for clinical decision-making. Treatment strategies informed by any of the 4 models are superior to the default strategies of treating all or no patients. The net benefit of the XGBoost model exceeded those of the other models at 20%-60% threshold probabilities.

### Model Interpretation

The XGBoost model, identified as the optimal model in terms of AUC value, was further explored for interpretation. Table 6 demonstrates the variable importance of the XGBoost model. Antepartum depression, TSH, fetal weight, serum ferritin, TPOAb, and age were the 6 variables that most influenced the outcome of the model.

**Figure 3.**
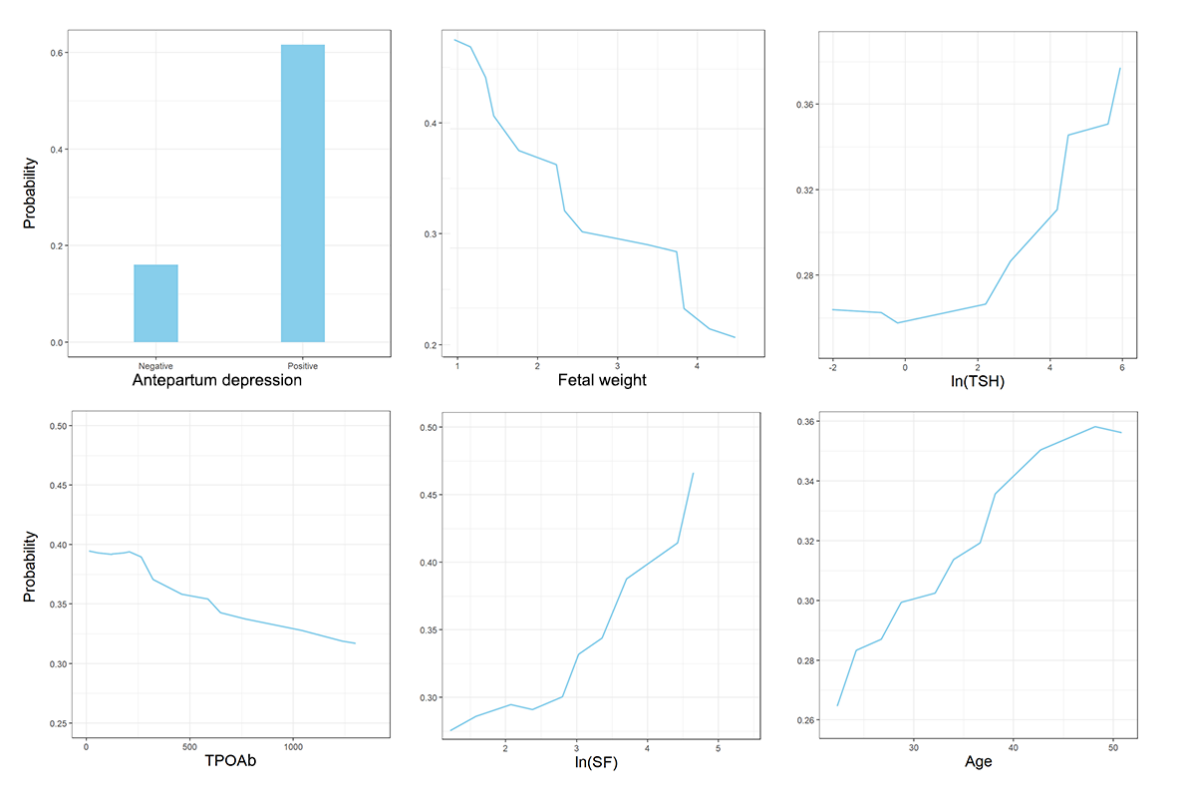
Partial dependence plots for the 6 most influential variables in the extreme gradient boosting model. The y-axis is set on a probability scale since our model was a binary classification model; the values of TSH and SF were on a logarithmic scale to present more pronounced trends in predicted probability as the values of the variables change. SF: serum ferritin; TPOAb: thyroid peroxidase antibody; TSH: thyroid-stimulating hormone.

PDPs illustrate a visual representation of the relationship between the most influential variables and the predicted response while accounting for the average effect of the other predictors in the model (Figure 3). As our predictive outcome is a binary categorical variable, the impacts of variables on the outcome were presented in the form of predictive probability ranging from 0 to 1. These plots indicate that the probability of PPD increases when participants have antepartum depression, higher TSH, higher serum ferritin, and older age. Likewise, the probability descends when participants have higher fetal weight and higher TPOAb.

**Table 6.** Variable importance for extreme gradient boosting model.

Variable	Importance
Prenatal depression	0.268
Fetal weight	0.169
TSH^a^	0.162
TPOAb^b^	0.132
Serum ferritin	0.131
Age	0.063
Apgar^c^ 10 minutes	0.024
Income	0.014
Occupation	0.012
Amniotic fluid volume disorder	0.011
Fetal distress	0.010
Family satisfaction	0.009
Only child	0.009
Intrauterine death	0.007
Rh negative	0.005
Adverse marital status	0.002
Ethnics	0.002

a TSH: thyroid-stimulating hormone.

b TPOAb: thyroid peroxidase antibody.

c Apgar: appearance, pulse, grimace, activity, and respiration.

**Figure 4.**
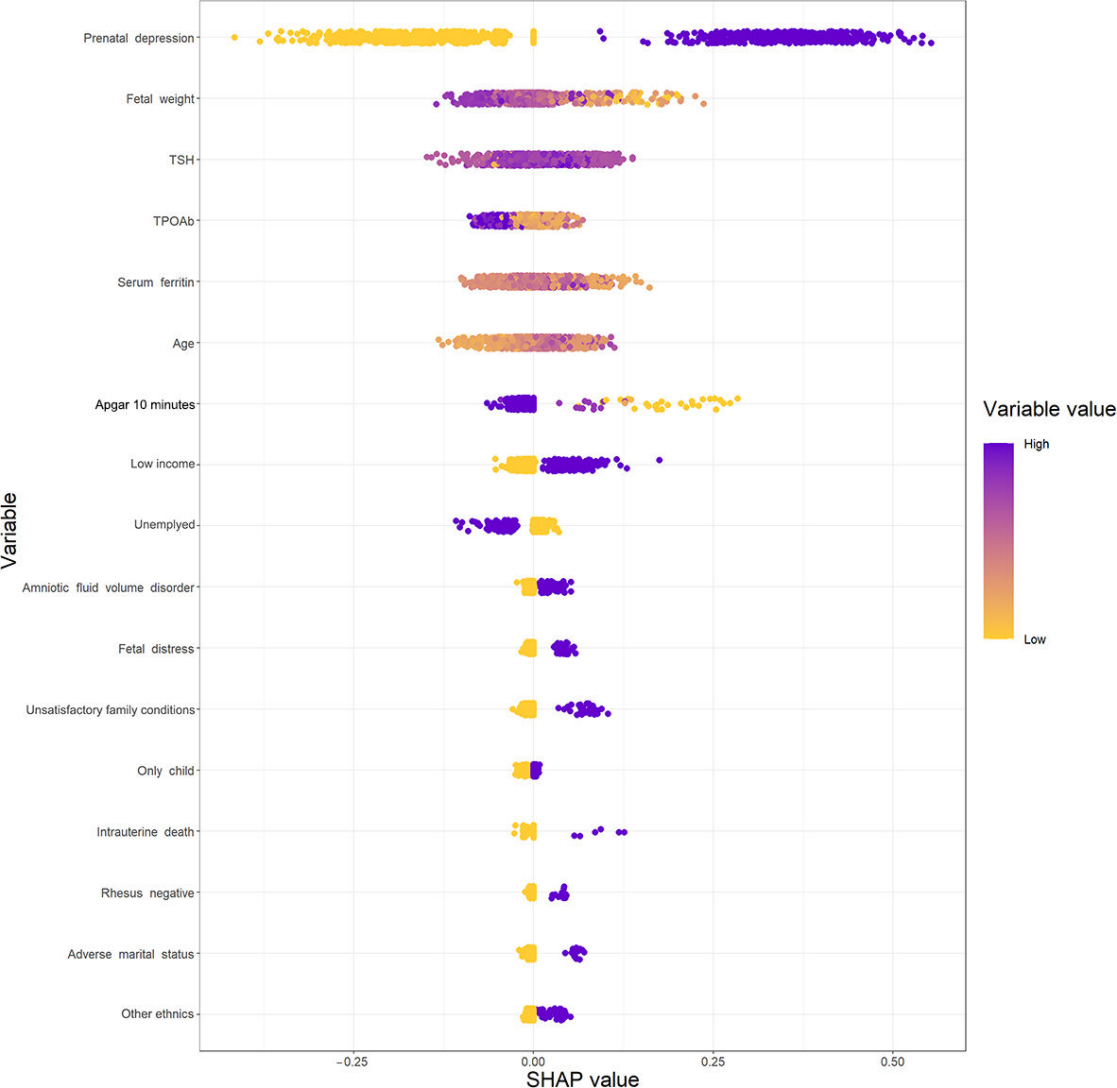
The SHAP values for the extreme gradient boosting model. Each dot in the figure represents a variable for a single participant. The horizontal position indicates whether that variable has a positive or negative impact on the predictive probability. Greater absolute values of SHAP represent a greater predictive probability of postpartum depression. The color shows the value of the variable for that observation. Purple indicates a higher value, representing the positive contribution of predictive outcome; and yellow indicates a lower value, representing the negative contribution of predictive outcome. A larger absolute value means that the variable has a greater impact on the result. For example, lower fetal weight (yellow dots) caused a negative impact on the predictive outcome. Since the SHAP system only accepts numeric input, binary categorical variables are converted to 0 (negative) and 1 (positive). In this case, low income and fetal distress are associated with a greater probability of postpartum depression. Apgar: appearance, pulse, grimace, activity, and respiration; SHAP: Shapley Additive Explanation; TPOAb: thyroid peroxidase antibody; TSH: thyroid-stimulating hormone.

SHAP provides an insight into how variables influence the prediction in each single sample (Figure 4). It can be concluded that the risk of PPD increases for participants with antepartum depression, lower fetal weight, lower level of TPOAb, elevated serum ferritin, and older age. The overall impact of TSH is not obvious. The interpretation of SHAP was mostly consistent with the interpretation of PDP. A higher risk of PPD is also associated with lower Apgar scores at 10 minutes, low income, amniotic fluid volume disorder, fetal distress, unsatisfactory family conditions, only child in the family, intrauterine death, Rh negative blood type, adverse marital status, and other ethnics.

## Discussion

### Principal Findings

This study developed and validated a machine learning–based model for prediction of PPD with an AUC of 0.849. Through the model interpretation of our optimal XGBoost model, several significant predictors of PPD were identified. These findings derived from the XGBoost model provide insightful contributions to the understanding of PPD.

Based on variable importance, antepartum depression was the most influential predictor of PPD in our analysis. Women with antepartum depression are likely to extend depressive symptoms into the postpartum period. In an epidemiology study, more than 54% of women with PPD reported depressive symptoms during pregnancy [5]. Several studies excluded participants with antepartum depression to focus on newly diagnosed PPD [16,43]. This might avoid the bias associated with chronic depression but neglect the impact of maternal prenatal mental status on PPD.

In addition, our study identified key biochemical markers of PPD including TSH, TPOAb, and serum ferritin. According to the result of the PDP interpretation, women with elevated serum ferritin levels were prone to PPD. Ferritin serves a critical role in the synthesis of monoamine neurotransmitters including dopamine [44]. With excessive ferritin, neurotransmitter dysregulation might play a part in the onset of PPD [45]. This finding warns the risks of excessive iron supplementation during pregnancy. Apart from that, elevated TSH and declined TPOAb were associated with a higher risk of PPD in our model interpretation. Elevated TSH often indicates hypothyroidism, whose clinical manifestation includes depression [46]. TPOAb is an autoantibody against the enzyme thyroid peroxidase and is commonly associated with autoimmune thyroid diseases such as Hashimoto thyroiditis [47,48]. A systematic review reported that the association between TPOAb and PPD remains controversial [49]. The specific mechanism of how TPOAb affects PPD requires more investigation.

Additionally, women with older age and lower infant weight were prone to PPD in our findings. This aligns with existing literature [49]. Other commonly recognized predictors like lack of social support, gestational diabetes, and overweight were excluded in the LASSO regression due to potential multicollinearity. These results not only validate some of the existing hypotheses about the pathophysiology of PPD but also open new avenues for research, particularly in the context of developing more effective, holistic screening methods.

Our study offers several advancements over previous research on PPD prediction. Earlier studies predominantly focused on clinical factors, such as obstetric history and comorbidities during pregnancy [50-52]. Our research comprehensively collected variables from multiple domains, including clinical, psychosocial, and biochemical markers. This broader scope allows for a more holistic view of the potential risk factors contributing to PPD, addressing the multifactorial nature of the condition. In addition, many previous studies were limited by relatively small sample sizes, often involving fewer than 1000 participants, which may have restricted the generalizability of their findings [50,51,53]. In contrast, our study included a larger cohort of over 2000 participants, providing greater statistical power and a more reliable basis for identifying significant risk factors. This larger sample size also enhances the model’s ability to detect more subtle associations between variables and PPD, which might have been overlooked in studies with smaller sample size. Moreover, previous research has often been limited to populations in Western countries [54,55]. Our study focuses on a Chinese cohort, offering insights that are culturally specific and potentially more relevant for addressing PPD in non-Western settings. This regional focus helps fill a critical gap in the literature, as the risk factors and prevalence of PPD may vary significantly between different cultural and geographical populations. Finally, our inclusion of biochemical markers, such as thyroid function and serum ferritin levels, adds a novel dimension to PPD research. These physiological indicators have rarely been incorporated in prior studies; yet, they may play a crucial role in understanding the biological underpinnings of PPD. By integrating these markers with traditional clinical and psychosocial factors, our study provides a more comprehensive framework for early detection and intervention.

Although our study was conducted on a specific population from Southwestern China, the comprehensive nature of the variables included in the model suggests that it could be adapted to other populations with similar characteristics. Future studies could explore the model’s applicability in different regions and cultural contexts, potentially leading to a robust, universally applicable tool for PPD prediction.

Our study offers significant insights into the clinical application of machine learning models for PPD. By integrating a broad spectrum of both biochemical and psychosocial factors, our models offer a more nuanced and accurate prediction compared to traditional methods. Incorporating often-overlooked indicators such as thyroid function and iron metabolism provides insight into the early screening of PPD. The XGBoost model, which demonstrated the highest performance, is particularly valuable for its ability to manage complex interactions between variables, making it universally applicable in a clinical setting where multiple risk factors are at play. This comprehensive approach enhances the understanding risk factors for PPD and supports more effective early interventions. Future research can build on these findings by validating them in larger, more diverse populations, integrating these predictive factors into routine prenatal care, and exploring interventions targeting these risks to reduce the incidence of PPD.

### Limitations

This work is not without limitations. First, our analysis is constrained by the retrospective nature of the data, which may introduce biases such as recall bias or selection bias. Additionally, during the process of variable screening, removing variables with collinearity ensured the independence of final predictors, but also eliminated substantial amounts of variables, leading to loss of information. Furthermore, the study’s reliance on a specific population from a single institution may limit the generalizability of our findings to broader, more diverse populations.

### Conclusions

This study developed and validated several machine learning–based models for predicting PPD, integrating a comprehensive set of clinical, psychosocial, and biochemical factors, and incorporating a larger sample size. The XGBoost model was considered as the optimal model with an AUC of 0.849. Interpretation derived from the predictive model revealed significant predictors of PPD, encompassing antepartum depression, elevated TSH, declined TPOAb, elevated serum ferritin, older age, and lower infant weight. These identified risk factors could be implemented to the early screening of PPD for individuals at high risk. These findings underscore the advantages of integrating diverse predictors and advanced machine learning techniques to improve early screening for PPD. This approach not only enhances prediction accuracy but also provides valuable insights for future research and clinical applications.

## Abbreviations

Apgar: appearance, pulse, grimace, activity, and respiration
AUC: area under the receiver operating characteristic curve
*CCMD-3*: *Chinese Classification and Diagnostic Criteria of Mental Disorders, Third Edition*
DCA: decision curve analysis
*DSM-5*: *Diagnostic and Statistical Manual of Mental Disorders, Fifth Edition*
GBM: gradient boosting machine
LASSO: least absolute shrinkage and selection operator
PDP: partial dependence plot
PPD: postpartum depression
RF: random forest
ROC: receiver operating characteristic curve
SHAP: Shapley Additive Explanation
TPOAb: thyroid peroxidase antibody
TSH: thyroid-stimulating hormone
VIF: variance inflation factor
XGBoost: extreme gradient boosting
